# Development and validation of a prognostic nomogram for gallbladder cancer patients after surgery

**DOI:** 10.1186/s12876-022-02281-2

**Published:** 2022-04-21

**Authors:** Xinsen Xu, Min He, Hui Wang, Ming Zhan, Linhua Yang

**Affiliations:** Department of Biliary-Pancreatic Surgery, Renji Hospital, School of Medicine, Shanghai Jiao Tong University, No. 160 Pujian Road, Shanghai, 200127 People’s Republic of China

**Keywords:** Gallbladder cancer, Prognosis, Nomogram, Model

## Abstract

**Background:**

Gallbladder cancer is associated with late diagnosis and poor prognosis. Current study aims to develop a prognostic nomogram for predicting survival of gallbladder cancer patients after surgery.

**Methods:**

Two large cohorts were included in this analysis. One consisted of 1753 gallbladder cancer patients from the Surveillance, Epidemiology, and End Results (SEER) database, and the other consisted of 239 patients from Shanghai Renji hospital. Significant prognostic factors were identified and integrated to develop the nomogram. Then the model was subjected to bootstrap internal validation and external validation.

**Results:**

Univariate and multivariate analysis indicated that age, tumor histology, T-stage, N-stage and M-stage were significant prognostic factors, which were all included to build the nomogram. The model showed good discrimination, with a concordance index (C-index) of 0.724 (95% CI, 0.708–0.740), and good calibration. Application of the nomogram in the validation cohort still presented good discrimination (C-index, 0.715 [95% CI 0.672–0.758]) and good calibration. In the primary cohort, the C-index of the nomogram was 0.724, which was significantly higher than the Nevin staging system (C-index = 0.671; *P* < 0.001) and the 8th TNM staging system (C-index = 0.682; *P* < 0.001). In the validation cohort, the C-index of the nomogram was 0.715, which was also higher than the Nevin staging system (C-index = 0.692; *P* < 0.05) and the 8th TNM staging system (C-index = 0.688; *P* = 0.06).

**Conclusions:**

The proposed nomogram resulted in more-accurate prognostic prediction for patients with gallbladder cancer after surgery.

## Introduction

Gallbladder cancer, the most common malignancy of the biliary tract, is usually associated with late diagnosis and poor prognosis [1]. Surgical resection remains the only definitively curative treatment. The most widely used staging system for gallbladder cancer is the Nevin staging system and American Joint Committee on Cancer (AJCC) TNM staging system [2, 3]. However, even within the same stage, the survival still varies widely.

Nomograms are graphical depictions of statistical models which quantify risks by incorporating significant variables [4, 5]. Currently, they have been widely applied in different types of cancer, which were reported to outperform the traditional staging systems for survival prediction [6–8]. With respect to gallbladder cancer, the nomogram was developed to evaluate the survival benefit of adjuvant radiotherapy and chemotherapy nearly 10 years ago [9, 10]. Until recently, Xiao et al*.* utilized the log odds of metastatic lymph node (LODDS) to predict the prognosis of gallbladder cancer [11]. Chen et al*.* also created a nomogram to predict overall survival for node-negative gallbladder cancer [12]. However, they only applied the 7th edition TNM staging system, rather than the most recently revised 8th edition TNM staging system. In addition, either the sample size of those studies was relatively small, or the study did not include external validation.

Hence, the aim of the present study was to develop and validate a practical nomogram that incorporated clinical and pathological factors for individual prediction of survival for patients with gallbladder cancer. Furthermore, we also determined whether this model provided more accurate survival prediction when compared with the Nevin staging system or the 8th edition TNM staging system.

## Methods

### Patients and data collection

The primary cohort of patients with gallbladder cancer was from the Surveillance, Epidemiology, and End Results (SEER) database (2004–2015) of the US National Cancer Institute. Initial patient selection was based on the SEER site recode “gallbladder” (International Classification of Diseases for Oncology, 3rd edition [ICD-O-3] site code C239). Only patients underwent surgical interventions with curative intent were included in the analysis. More specifically, at least a simple cholecystectomy or any more extensive surgery was performed, which was in accord with the “RX Summ-Surg Prim Site” codes between 30 and 90. Since the T stage and M stage definitions were almost the same across 6th, 7th and 8th TNM staging systems, the 8th edition TNM information was retrieved using the following codes: derived AJCC T 7th edition, derived AJCC M 7th edition, derived AJCC T 6th edition, derived AJCC M 6th edition, regional nodes positive and regional nodes examined. Exclusion criteria were as follows: unknown TNM information; unknown tumor size; unknown tumor grade; unknown tumor histology.

**Fig. 1:**
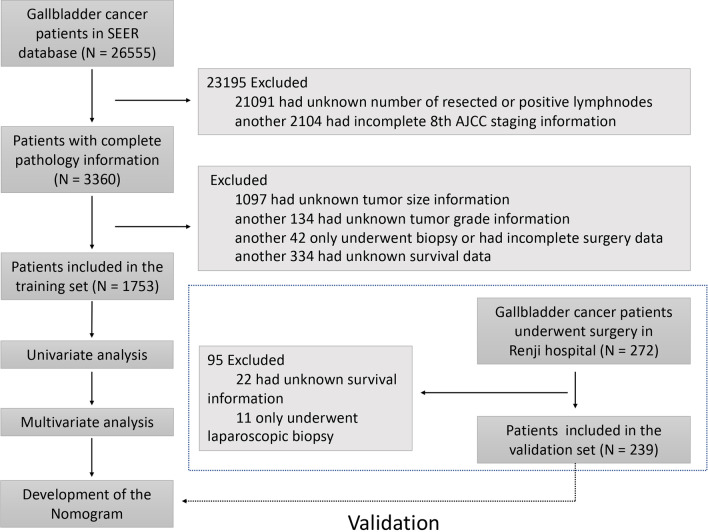
Flowchart showing patient selection process and study workflow

The validation cohort of gallbladder cancer patients who underwent surgical resection was from the Renji hospital, Shanghai Jiaotong University (Renji dataset; 2010–2018). This study was conducted in accordance with the Declaration of Helsinki. Informed consent was obtained from all subjects and/or their legal guardians. This study was approved by the Ethical Committee of Renji hospital, Shanghai Jiao Tong University. Patients with missing data regarding tumor pathology or overall survival were excluded. Patients who only underwent laparoscopic biopsy were also excluded. A flowchart of patient selection and study workflow was shown in Fig. 1.

### Statistical analysis

Statistical analysis was conducted by the R software (version 3.6.2). The primary end point in this study was overall survival (OS). Observed variables included age, sex, surgery type, tumor number, size, grade, histology, AJCC TNM stage information, number of positive lymph nodes and number of resected lymph nodes. Continuous variables were transformed into the categorical variables based on the routine cut-off value. Kaplan–Meier curves were constructed to evaluate the overall survival of patients in different AJCC TNM stages, and the log-rank test was performed.

Significant variables calculated from the univariable and multivariate analysis were selected to build the nomogram, which was subjected to bootstrapping validation (1,000 bootstrap resamples). Prognostic performance was evaluated by the concordance index (C-index). Calibration of the nomogram was performed by comparing the predicted 1-, 3-, and 5-year OS with actual survival. In addition, a receiver operating characteristic (ROC) curve and the area under curve (AUC) was calculated to evaluate the accuracy. With respect to the external validation, total points of each patient in the validation cohort were calculated based on the nomogram, and cox regression was performed to obtain the C-index. A *P* value of less than 0.05 was considered statistically significant.

## Results

### Patient characteristics

**Fig. 2:**
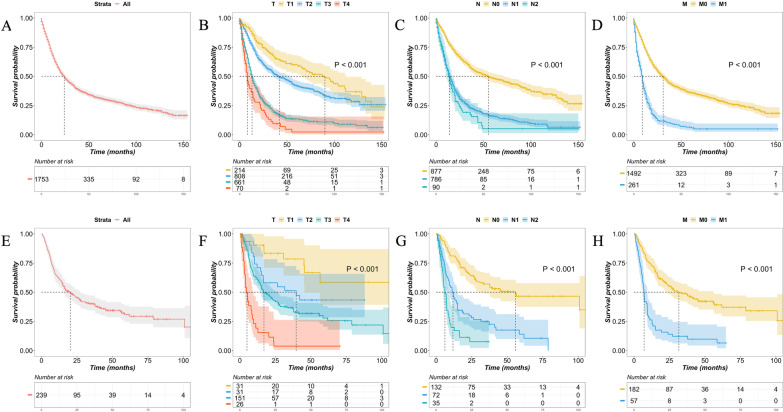
Kaplan–Meier survival curves of the gallbladder cancer after surgery. **A** Survival curves of the SEER cohort; **B** survival curves stratified by 8th edition AJCC pathologic T category of the SEER cohort; **C** survival curves stratified by 8th edition AJCC pathologic N category of the SEER cohort; **D** survival curves stratified by 8th edition AJCC pathologic M category of the SEER cohort; **E** survival curves of the Renji cohort; **F** survival curves stratified by 8th edition AJCC pathologic T category of the Renji cohort; **G** survival curves stratified by 8th edition AJCC pathologic N category of the Renji cohort; **H** survival curves stratified by 8th edition AJCC pathologic M category of the Renji cohort

Of the 26,555 gallbladder cancer patients in the SEER database (training set), 21,091 patients with unknown number of resected or positive lymph nodes and 2104 patients with incomplete 8th AJCC TNM staging information were excluded. Patients who had missing values on any of the examined variables, including tumor size (n = 1097), tumor grade (n = 134) and survival information (n = 334), were also excluded. In addition, 42 patients who only received biopsy or had incomplete surgery data were removed from analysis. Thus, a total of 1753 patients were included in the analysis according to the inclusion and exclusion criteria, with a median survival of 24 months [95% confidence interval (CI), 22 to 27 months, Figs. 1 and 2]. The Renji database (validation set) consisted of 272 patients with gallbladder cancer diagnosed between 2010 and 2018, of which 22 had unknown survival information and 11 only received laparoscopic biopsy. Thus, a total of 239 patients were included in the analysis, with a median survival of 20 months (95% CI 15–28 months, Figs. 1 and 2). The clinicopathologic characteristics of patients in the SEER cohort and Renji cohort were listed in Table 1.

**Table 1:** Demographics and clinicopathologic characteristics of the SEER dataset (training set) and Renji dataset (validation set)

Demographic or clinicopathologic characteristic	SEER dataset (N = 1753)				Renji dataset (N = 239)			
	No. of patients	%	OS (months)		No. of patients	%	OS (months)	
			Median	95% CI			Median	95% CI
Sex								
Male	527	30	22	19–27	80	33	18	11–38
Female	1226	70	25	22–28	159	67	21	15–32
Age, years								
≥ 70	771	44	18	16–21	99	41	21	16–38
< 70	982	56	30	26–35	140	59	16	13–37
Resection type								
Radical resection	384	22	21	17–26	196	82	28	20–45
Palliative resection	1369	78	25	23–29	43	18	7	4–9
Tumor histology								
Papillary histology	114	6	89	78–NA	7	3	NA	7–NA
Squamous histology	80	5	10	9–17	18	8	9	5–NA
Adenocarcinoma and others	1559	89	23	21–25	214	89	21	15–28
Tumor grade								
I	237	13	40	33–88	23	9.6	NA	45–NA
II	788	45	32	27–36	158	66	17	14–28
III	679	39	15	14–18	57	24	14	8–23
IV	49	3	10	6–17	1	0.4	NA	NA
Tumor size, cm								
< 3	763	44	35	30–41	83	35	33	17–NA
3–5	534	30	21	18–25	87	36	26	17–42
5–10	393	22	15	14–19	60	25	9	8–17
≥ 10	63	4	10	8–14	9	4	7	6–NA
Tumor number								
Single tumor	1647	94	22	20–25	182	76	28	22–45
Multiple tumors	106	6	57	37–72	57	24	7	6–10
Pathologic T category								
T1	214	12	90	66–112	31	13	NA	45–NA
T2	808	46	42	35–53	31	13	40	15–NA
T3	661	38	13	12–14	151	63	17	14–27
T4	70	4	8	7–14	26	11	5	3–10
Pathologic N category								
N0	877	50	55	45–72	132	55	56	31–NA
N1	786	45	14	13–16	72	30	12	9–15
N2	90	5	14	10–17	35	15	6	5–9
Pathologic M category								
M0	1492	85	31	28–35	182	76	32	23–56
M1	261	15	9	8–10	57	24	7	6–9
8th AJCC stage								
I	180	10	99	87–139	29	12	NA	56–NA
II	408	23	90	78–127	18	8	NA	26–NA
III	855	49	19	17–20	113	47	28	21–56
IV	310	18	9	8–10	79	33	7	6–9

*NA* not available

### Significant prognostic factors in the training set

The 1-, 3-, and 5-year OS of the SEER patients were 67.7%, 39.9% and 31.9%, respectively (Fig. 2A). The results of the univariate analysis were shown in Table 2. Age, tumor size, tumor grade, pathologic T category, N category and M category were demonstrated to be significantly associated with overall survival (*P* < 0.001). Among all cell types, papillary histology had the most favorable survival, followed by adenocarcinoma and squamous cell carcinoma (*P* < 0.001). Interestingly, the tumor number was shown to be a favorable factor for prognosis in the SEER patients, which was in contrast with the common sense and the result from the Renji cohort, that patients with more tumors had worse survival outcome. Thus, all significant factors but the tumor number in the univariate analysis were entered into the multivariate analysis based on the cox regression. Results showed that age, tumor histology, pathologic T category, N category and M category remained the significant prognostic factors by the multivariable analysis (*P* < 0.001).

**Table 2:** Univariate analysis, multivariate analysis and cox regression analysis for building the model

Variable	Univariate analysis P	Multivariate anlaysis			Selected factors for building model		
		Hazard ratio	95% CI	P	Hazard ratio	95% CI	P
Age, years	< 0.001			< 0.001			< 0.001
≥ 70		Reference			Reference		
< 70		1.733	1.536–1.956	< 0.001	1.745	1.547–1.968	< 0.001
Tumor histology	< 0.001			< 0.001			< 0.001
Papillary histology		Reference			Reference		
Adenocarcinoma and others histology		1.667	1.236–2.248	< 0.001	1.684	1.251–2.266	< 0.001
Squamous histology		2.111	1.410–3.161	< 0.001	2.282	1.536–3.390	< 0.001
Tumor grade	< 0.001			0.469			
I		Reference					
II		0.997	0.814–1.221	0.975			
III		1.103	0.895–1.360	0.358			
IV		1.103	0.759–1.603	0.607			
Tumor size, cm	< 0.001			0.156			
< 3		Reference					
3–5		1.116	0.968–1.287	0.131			
5–10		1.133	0.965–1.331	0.127			
≥ 10		1.343	0.989–1.825	0.059			
Pathologic T category	< 0.001			< 0.001			< 0.001
T1		Reference			Reference		
T2		0.994	0.783–1.263	0.963	1.003	0.790–1.273	0.982
T3		1.960	1.530–2.510	< 0.001	2.064	1.619–2.632	< 0.001
T4		2.244	1.570–3.206	< 0.001	2.384	1.682–3.381	< 0.001
Pathologic N category	< 0.001			< 0.001			< 0.001
N0		Reference			Reference		
N1		2.024	1.770–2.316	< 0.001	2.076	1.818–2.370	
N2		2.026	1.557–2.637	< 0.001	2.086	1.606–2.709	
Pathologic M category	< 0.001			< 0.001			< 0.001
M0		Reference			Reference		
M1		2.242	1.915–2.626	< 0.001	2.300	1.969–2.687	< 0.001
Sex	0.083						
Male							
Female							
Resection type	0.062						
Radical resection							
Palliative resection							
Tumor number	< 0.001*						
Single tumor							
Multiple tumors							

^*^The tumor number was excluded in the multivariable analysis, since its hazard ratio was less than 1, which was contrary to the factor that patients with more tumors had worse survival outcome

### Prognostic nomogram for OS

**Fig. 3:**
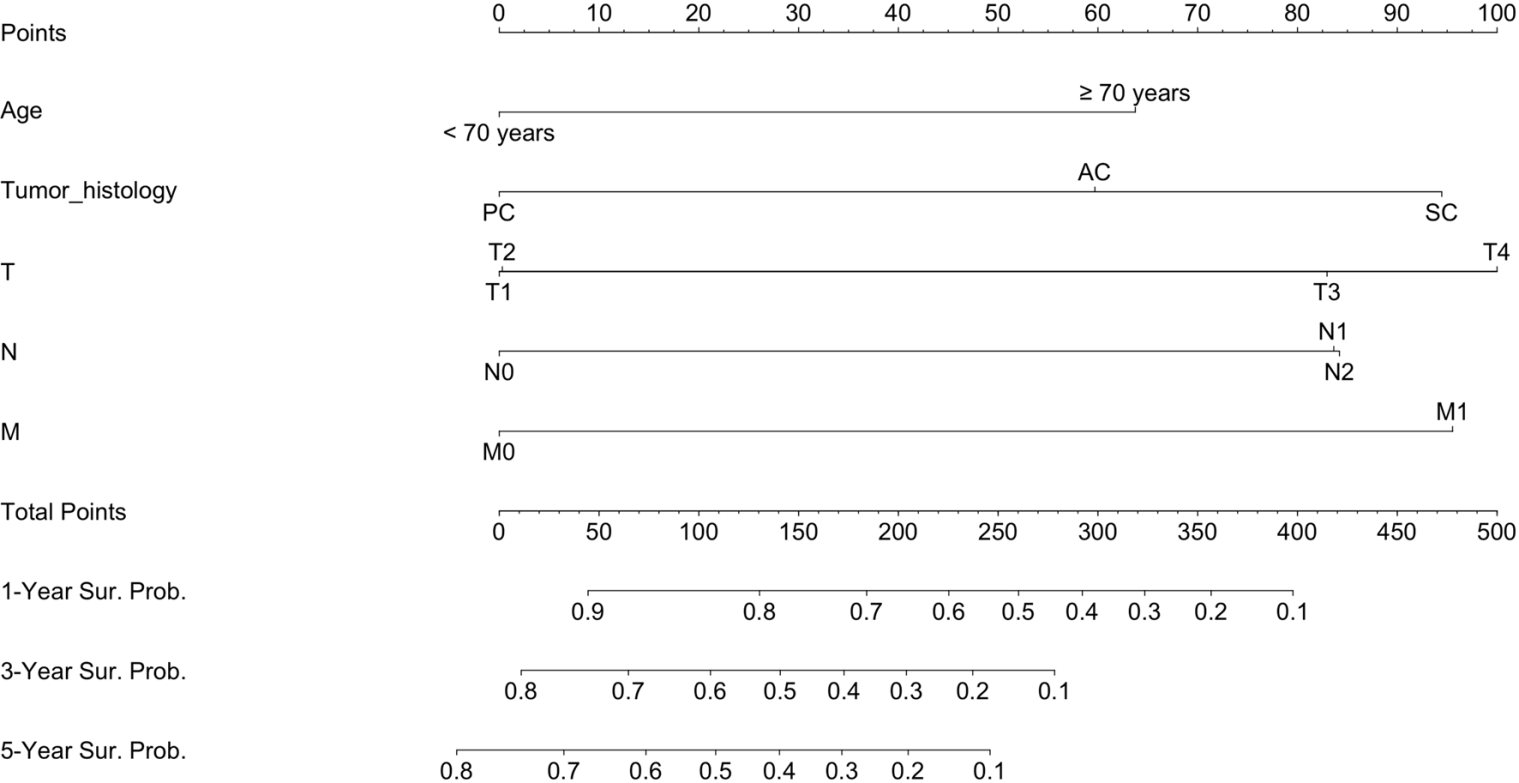
Developed nomogram for gallbladder cancer survival after surgery. *PC* papillary carcinoma, *AC* adenocarcinoma, *SC* squamous cell carcinoma

**Fig. 4:**
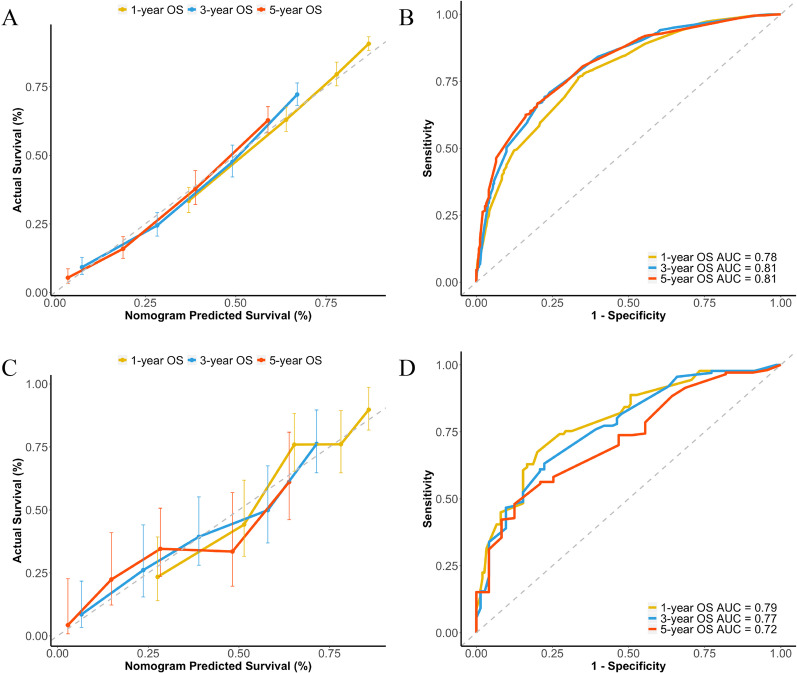
Calibration curves demonstrating the agreement between the nomogram predicted survival and actual survival, and ROC curves demonstrating the prediction accuracy. **A** Calibration curves for 1-, 3-, 5-year OS of the SEER cohort; **B** ROC curves for 1-, 3-, 5-year OS of the SEER cohort; **C** Calibration curves for 1-, 3-, 5-year OS of the Renji cohort; **D** ROC curves for 1-, 3-, 5-year OS of the Renji cohort

The prognostic nomogram integrating all significant factors for OS in the training set was shown in Fig. 3. The calibration plots for the probability of 1-, 3-, and 5-year OS showed an optimal agreement between the nomogram predicted survival and actual survival (Fig. 4A). The C-index of the nomogram was 0.724 (95% CI, 0.708 to 0.740), and the AUC values of the ROC curves at the 1-, 3-, and 5-year OS were 0.78, 0.81 and 0.81, respectively (Fig. 4B).

In the validation set, the calibration plots for the probability of 1-, 3-, and 5-year OS also showed an optimal agreement between the nomogram predicted survival and actual survival (Fig. 4C). The C-index of the nomogram was 0.715 (95% CI, 0.672 to 0.758), and the AUC values of the ROC curves at the 1-, 3-, and 5-year OS were 0.79, 0.77, and 0.72, respectively (Fig. 4D).

### Comparison of predictive accuracy between nomogram and conventional staging systems

**Fig. 5:**
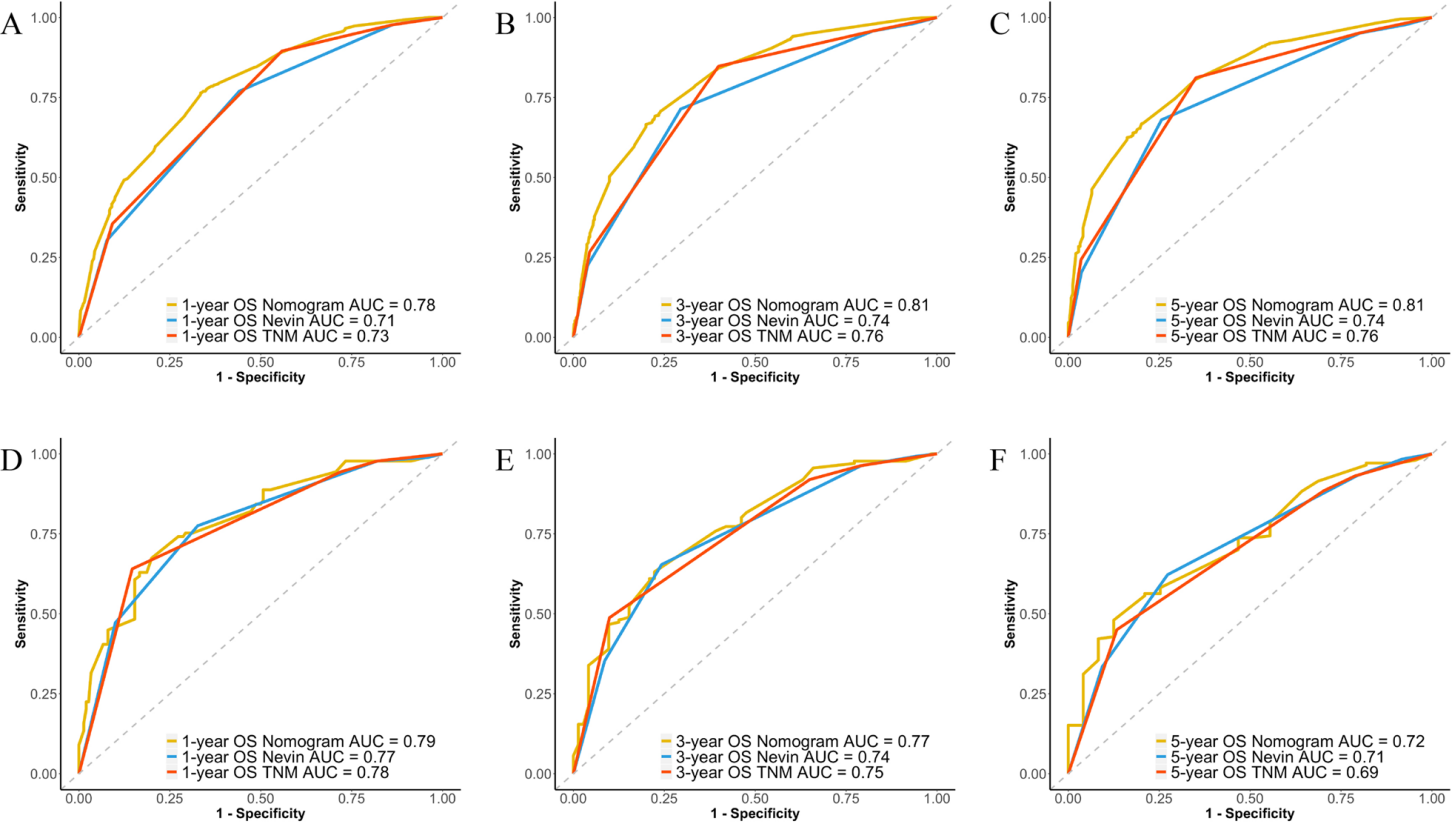
ROC curves comparing the prediction accuracy among the nomogram, Nevin staging system and 8th AJCC TNM staging system. **A** ROC curves for 1-year OS of the SEER cohort; **B** ROC curves for 3-year OS of the SEER cohort; **C** ROC curves for 5-year OS of the SEER cohort; **D** ROC curves for 1-year OS of the Renji cohort; **E** ROC curves for 3-year OS of the Renji cohort; **F** ROC curves for 5-year OS of the Renji cohort

The most widely used staging system for gallbladder cancer is the Nevin staging system and 8th American Joint Committee on Cancer (AJCC) TNM staging system. Our results showed that the nomogram displayed better accuracy in predicting overall survival in both the training set and validation set (Fig. 5). In the training set, the C-index of the nomogram was 0.724, which was significantly higher than the Nevin staging system (C-index = 0.671; *P* < 0.001) and the 8th TNM staging system (C-index = 0.682; *P* < 0.001). In the validation set, the C-index of the nomogram was 0.715, which was also higher than the Nevin staging system (C-index = 0.692; *P* < 0.05) and the 8th TNM staging system (C-index = 0.688; *P* = 0.06).

Xiao et al. reported that log odds of metastatic lymph node (LODDS), defined as the ratio of the number of positive lymph nodes to the number of negative nodes, was the best N stage in the SEER database. We also tested that whether the nomogram could display better accuracy when replacing the 8th AJCC N stage with LODDS in the nomogram. Consistent with the results demonstrated by Xiao et al., the C-index of the nomogram increased from 0.724 to 0.733 in the SEER database (*P* < 0.001). However, on the contrary, the C-index decreased from 0.715 to 0.708 in the validation set (*P* > 0.05). There’s no obvious improvement in the prediction of overall survival in the Renji dataset.

## Discussion

Gallbladder cancer is remarkably heterogeneous with respect to survival of individual patients. Although the Nevin staging system and AJCC TNM staging system are widely used, the prediction accuracy is still far from satisfactory. Identifying patients with varied survival outcomes will be beneficial for clinical decision making.

In our study, age, histology, pathologic T category, N category and M category were identified as significant prognostic factors, which were consistent with previous reports [9, 11–14]. We have previously shown that age was a significant prognostic factor in liver cancer [15]. Similarly, age was also demonstrated to be inversely correlated with survival in many different cancer types, which was a common prognostic factor for cancer [5, 7]. As for gallbladder cancer histology, Sandeep et al*.* previously demonstrated that gallbladder papillary carcinoma had the best survival outcomes, followed by adenocarcinoma, while squamous carcinoma had the worst survival outcomes, which was consistent with our results [14].

The gallbladder cancer pathologic T category, N category and M category were common significant prognostic factors for building the nomogram [11, 12]. However, the definitions have been modified for several times by the AJCC. In the 6th, 7th and 8th edition, the tumors with more than 2 cm invasion into the liver were reclassified from T4 as T3. In addition, a new T4 category was added for tumors invading portal vein, hepatic artery, or multiple extrahepatic organs. Notably, a major change of N category was made in the most recent 8th edition, that N category was based on the number of positive lymph nodes, rather than the location of the positive lymph nodes. In the 8th edition, more focus was put to the number of lymph node metastasis, as our previous study also proposed that extended lymphadenectomy might better ensure the R0 resection than the regional lymphadenectomy [16]. To the best of our knowledge, this is the first nomogram study of gallbladder cancer based on the 8th edition AJCC TNM staging system. Furthermore, compared with previous built models, our model was based on the largest sample size, both in the training cohort and validation cohort [11, 12].

Previously, Xiao et al. reported that LODDS was the best N stage in the SEER database [11]. However, there’s no obvious improvement in the prediction of overall survival in our validation cohort. We suppose that inadequate number of lymph nodes dissection might lead to the bias, especially when laparoscopic cholecystectomy was performed for the incidental gallbladder cancer, and when palliative excision was performed for the stage IV patients.

In this study, the C-index of the nomogram was higher than that of the Nevin staging system and the 8th edition TNM staging system for predicting overall survival, suggesting the preponderance of the nomogram over the widely used staging systems. In addition, the nomogram only incorporated age, tumor histology, pathologic T category, N category and M category, which was convenient to use for both clinicians and patients.

There were several limitations of this study. Firstly, the SEER database did not provide blood test data for the patients, such as blood routine test, liver function and tumor markers. These serum factors might help improve the prediction accuracy of the nomogram. Secondly, the sample size of the validation cohort was relatively small. Future studies of prospective, randomized and multicenter trials are needed to modify the nomogram. Thirdly, genomics data and radiomics data, which represent the precision medicine, are encouraged to be incorporated to improve this model [17].

## Conclusion

In conclusion, we established and validated a novel nomogram which resulted in more-accurate prognostic prediction for patients with gallbladder cancer after surgery. Through this model, patients might estimate the survival more precisely, and clinicians could better identify patients with different death risks and provide treatment options.

## Data Availability

The authors declare that all data supporting the findings of this study are available within the main text, or from the corresponding author on reasonable request.

## Abbreviations

SEER: Surveillance, epidemiology, and end results
C-index: Concordance index
AJCC: American Joint Committee on Cancer
LODDS: Log odds of metastatic lymph node
ICD-O-3: International Classification of Diseases for Oncology, 3rd edition
OS: Overall survival
ROC: Receiver operating characteristic
AUC: Area under curve
